# The feasibility and diagnostic accuracy by multiple cardiac biomarkers in emergency chest pain patients: a clinical analysis to compare 290 suspected acute coronary syndrome cases stratified by age and gender in Taiwan

**DOI:** 10.1186/s12872-016-0374-4

**Published:** 2016-10-07

**Authors:** Chung-Lieh Hung, Ding-Kuo Chien, Shou-Chuan Shih, Wen-Han Chang

**Affiliations:** 1 Department of Internal Medicine, Division of Cardiology, MacKay Memorial Hospital, Taipei, Taiwan; 2 Department of Medicine, Mackay Medical College, New Taipei city, Taiwan; 3 Emergency Department, MacKay Memorial Hospital, Taipei, Taiwan; 4 Department of Nursing, Mackay Junior College of Medicine, Nursing and Management, Taipei, Taiwan; 5 Graduate Institute of Injury Prevention and Control, Taipei Medical University, Taipei, Taiwan; 6 Institute of Mechatronic Engineering, National Taipei University of Technology, Taipei, Taiwan; 7 Department of Internal Medicine, Division of Gastroenterology, MacKay Memorial Hospital, Taipei, Taiwan; 8 92, Section 2, Chungshan North Road, Taipei, Taiwan; 9 School of Medicine, Taipei Medical University, Taipei, Taiwan

**Keywords:** Multiple cardiac biomarker, Acute coronary syndrome, Elderly

## Abstract

**Background:**

Accurate diagnosis of acute coronary syndrome (ACS) in a timely fashion is challenging in the elderly population, especially elderly women, who usually exhibit atypical clinical symptoms. A multiple cardiac biomarker (MCB) based approach has been shown to improve diagnostic efficacy of ACS. However, data in various age groups and sex differences remain largely unexplored.

**Methods:**

Point-of-care testing (POCT) was performed on 290 patients (aged ≥18 years) who were admitted to the emergency department (ED) with symptoms of acute chest pain under suspicion of acute coronary syndrome (ACS). The MCB approach in current work assessed four cardiac biomarkers: myoglobin, troponin I, creatine kinase-myocardial band isoenzyme fraction (CK-MB), and brain natriuretic peptide (BNP).

**Results:**

Overall, the MCB approach demonstrated considerably higher sensitivity for elderly patients than for younger patients in identifying ACS (80.0 % [64.1–90.0] vs. 52.6 % [37.3–67.5] for ≥65 years and <65 years groups), with younger population showed greater specificity (44.1 % [35.3–53.4] vs. 84.9 % [76.9–90.5] for ≥65 years and <65 years groups, respectively). The highest sensitivity achieved for elderly women who reported chest pain was 87.5 % [95 % CI: 64–96.5]). In general, the sensitivity of this approach was higher for female patients than for male patients (80 % [58.4–91.9] vs. 61 % [47.8–73.0]).

**Conclusions:**

The MCB approach can provide a quick and accurate clinical diagnosis in elderly and female patients, both of whom have traditionally proven to be challenging to diagnose from suspected acute coronary syndrome.

## Background

Acute coronary syndrome (ACS) is an extremely common condition that is one of the leading causes of death worldwide. ACS is commonly encountered in the emergency room and occurs most often in elderly patients [1, 2]; however, in recent years the average age of patients with ACS has declined due to changes in lifestyle and personal behavior.

Women who have ACS differ from their male counterparts both in terms of reported symptoms, clinical manifestations and diagnosis rates [3–5]. In terms of symptoms and clinical manifestations, female patients primarily exhibit unstable angina and non-ST-segment elevation myocardial infarction (NSTEMI), while male patients primarily exhibit STEMI [3, 5]. Regarding diagnosis, biomarker sensitivity and specificity in exercise tests have been reported to be lower in both women and men [5].

Cardiac biomarkers for acute coronary syndrome play key roles diagnosis, risk assessment, treatment, and supervision. Troponin I is a sensitive indicator of cardiomyocyte necrosis and an important biomarker for monitoring treatment. Myoglobin is the earliest biomarker of myocardial injury to appear, allowing doctors to rule out the possibility of myocardial infarction in a timely fashion. Apart from diagnostic role in heart failure, brain natriuretic peptide (BNP) is also closely related to acute coronary syndrome [6]. However, when used individually, these biomarkers are limited by several factors in clinical scenarios, especially in elderly patients [2].

Owing to differences in the diagnostic ability and performance of cardiac biomarkers across a wide range of patient population with age and gender distributions, and considering their important role in the diagnosis of acute coronary syndrome, we currently examined the accuracy of four cardiac biomarkers in the diagnosis of acute coronary syndrome in patients who experienced chest pain.

## Methods

### Patient enrollment

From March to November 2009, patients who visited Linkou Chang Gung Memorial Hospital, Taipei Mackey Memorial Hospital, Far Eastern Memorial Hospital, and China Medical University Hospital in Taiwan, with a primary complaint of chest pain and who were suspected of having acute coronary syndrome were recruited into this study. All enrolled patients were aged 18 years or older. Inclusion criteria includes: 1) adults ≥18 years of age and consented to participate in the study group, 2) admitted to the emergency room due to chest pains or potential acute coronary syndrome symptoms, 3) symptoms unclear or atypical but suspected to be ACS requiring further electrocardiogram analysis and blood tests. Exclusion criteria includes: 1) Pregnancy, or clinical scenarios that may cause false positive test results of elevated Troponin I or BNP, such as skeletal myopathies, or renal insufficiency [7, 8] (defined by creatinine >2.5 mg/dL), 2) Non-Taiwan residents, patients whose contact information is unavailable, patients planning to go abroad and thus would be unavailable for follow-up, and patients who had already spent more than two hours in the emergency room reception area, 3) Patients who were transferred from other hospitals, 4) Cases where written permission could not be obtained or refused to participate the study, 5) Cases in whom the symptoms were already confirmed as not the cause of acute coronary syndrome, including pneumothorax, acute pulmonary embolism, aortic dissection, acute gastroenterological diseases or chronic obstructive lung disorder, 6) Initial electrocardiogram (ECG) had shown elevated ST segment or new LBBB. Subjects with ST elevation and new left bundle branch block (LBBB) were excluded since these clearly showed the onset of AMI, and therefore did not require diagnosis by MCB test systems. Researchers then examined patients’ medical records and conducted telephone interviews to identify any subsequent health issues or discomfort experienced within 45 days after diagnosis. Within 45 days of treatment, cardiologists diagnosed patients with one of the following conditions as positive for acute coronary syndrome: ST-segment elevation myocardial infarction (STEMI); non-ST segment elevation myocardial infarction (NSTEMI); sudden cardiac arrest, high-grade atrioventricular block (AVB) or ventricular arrhythmia with cardiac catheter-proven coronary occlusion by thrombus formation; emergency, urgent, or elective revascularization; and unstable angina. All study participants underwent cardiac catheter during admission to confirm the diagnosis of acute coronary syndrome by experienced interventionalists/cardiologists and patients without these conditions were not enrolled in the study.

### Biochemical analysis and multi cardiac biomarker panel test

During the examination, myoglobin, creatine kinase-myocardial band isoenzyme fraction (CK-MB), troponin I, and BNP were tested using a MCB CardioProfiler Panel (Alere, San Diego, CA). Blood (3 ml) was sampled from each patient using EDTA K2 Vacuette tubes. Next, 0.25 ml of blood was siphoned from the total sample using a pipette and placed onto a test panel. The MCB test panel was then placed into a Triage MeterPro (Alere, San Diego, CA) for examination, the results of which were automatically displayed and printed after 15 min. Data were presented in quantitative form with continuous variables presented. All results were blinded to investigators. Biomarker reference values (CardioProfiler Panel) were as follows: Troponin *I* > 0.4 ng/mL, CK-MB > 4.3 ng/mL, myoglobin > 107 ng/mL, and BNP > 100 pg/mL.

### Statistical analysis

Quantitative data were expressed as mean ± standard deviation. Parametric data were compared by *t* test, and categorical data were analyzed by the Chi-square test with Yates’ correction or Fisher’s exact test. Data were analyzed using SPSS® statistics software (SPSS 11.5; SPSS Inc., Chicago, IL, USA). Data were analyzed for sensitivity, specificity, positive and negative predictive values (PPV and NPV), and positive and negative likelihood ratios (LR+ and LR−). All *P* < 0.05 was considered statistically significant with two-sided analysis with *P* < 0.05 was considered statistically significant.

## Results

A total of 290 patients with chest pain were enrolled in the current study with an average age of 64.5 years. Of the 290 patients, 146 were elderly (≥65 years), with an average age of 75.4 years, which represented 50.3 % of the total sample. The remaining 144 patients were <65 years of age (average age: 53.4 years) which represented 49.7 % of the total sample. Further, 109 patients were women (71 elderly and 38 young), and constituted 37.6 % of the total sample (Table 1).

**Table 1:** The statistics of characters between the elderly group and younger group

Patient’s group Variables	All	Patients ≥65 years	Patients <65 years	*P* value
Number	290	146	144	
Age (in years)	64.49 ± 13.1	75.4 ± 6.6	53.4 ± 7.6	
Female	109 (37.6 %)	71 (48.6 %)	38 (26.4 %)	
Cardiovascular Event History				
Myocardial infarction	40 (13.8 %)	21 (14.4 %)	19 (13.2 %)	0.41
Angina pectoris	87 (30 %)	44 (30.1 %)	43 (29.9 %)	0.48
Tachycardia	4 (1.4 %)	2 (1.4 %)	2 (1.4 %)	0.49
Cardiovascular diseases*	100 (34.5 %)	57 (39 %)	43 (29.8 %)	<0.05
Cardiac arrhythmia	19 (6.5 %)	12 (8.2 %)	7 (4.9 %)	0.18
Congestive heart failure*	15 (5.2 %)	13 (8.9 %)	2 (1.4 %)	<0.05
Stroke or temporal ischemia*	20 (6.9 %)	16 (10.9 %)	4 (2.8 %)	<0.05
Coronary artery bypass grafting surgery	9 (3.1 %)	6 (4.1 %)	3 (2.1 %)	0.27
Coronary angioplasty	54 (22.5 %)	30 (20.5 %)	24 (16.7 %)	0.35
Risk Factor				
High blood pressure*	176 (60.7 %)	103 (70.5 %)	73 (50.7 %)	<0.05
Diabetes*	89 (30.7 %)	53 (36.3 %)	36 (25 %)	<0.05
Lipidemia*	89 (30.7 %)	36 (24.6 %)	53 (36.8 %)	<0.05
Family history of Cardiovascular disease*	68 (23.4 %)	23 (15.7 %)	45 (31.2 %)	<0.05
Smoker*	64 (22.1 %)	7 (4.8 %)	57 (39.6 %)	<0.05

Data: mean (standard deviation), number (percentage)

*mean *P* < 0.05

A prior history of cardiovascular diseases (including prior stroke, myoacrdial infarction, history of coronary interventions or bypass graft surgery) was reported in 34.5 % of the participants (100/290). Other common comorbidities included angina (30 % [87/290]) and coronary angioplasty (22.5 % [54/290]). At baseline, elderly patients reported statistically significantly more medical conditions such as myocardial infarction, cardiovascular disease, heart failure, and stroke, compared with young patients (*p* < 0.05).

High blood pressure was found to be the most prevalent risk factor in patients with chest pain (60.7 %), followed by diabetes (30.7 %), and dyslipidemia (30.7 %). A larger proportion of elderly patients had high blood pressure and diabetes, while younger patients were more likely to have dyslipidemia, a family history of cardiovascular disease, or a history of smoking (Table 1).

A total of 73 (25.2 %) patients were diagnosed with cardiovascular disease, 35 of which were elderly (23.9 %) and 38 were young (26.4 %). Using the MCB approach in the diagnosis of acute coronary syndrome, young patients demonstrated a higher relative risk for acute coronary syndrome than elderly patients (6.25 vs. 3.16) (Table 2). In addition, 20 out of 109 (18.3 %) women were diagnosed with 53 out of 181 men (29.3 %). The MCB approach in the diagnosis of acute coronary syndrome revealed a higher relative risk in women than in men (6.17 vs. 3.36) (Table 3).

**Table 2:** Relation between MCB tests and Cardiovascular Events by Age

Age (Patient Number)				
Acute coronary syndrome (ACS)	≧65 years (*N* = 146)		<65 years (*N* = 144)	
	Positive	Negative	Positive	Negative
MCB positive	28 (19.2 %)	62 (42.4 %)	20 (13.9 %)	16 (11.1 %)
MCB negative	7 (4.8 %)	49 (33.5 %)	18 (12.5 %)	90 62.5 %)
Odds ratio	3.161*		6.25*	

* mean *P* < 0.05

**Table 3:** Relation between MCB tests and Cardiovascular Events By Gender

Gender (Patient Number)				
Acute coronary syndrome (ACS)	Male (*N* = 181)		Female (*N* = 109)	
	Positive	Negative	Positive	Negative
Cardiac biomarker positive	32 (17.6 %)	44 (24.3 %)	16 (14.6 %)	35 (32.1 %)
Cardiac biomarker negative	21 (11.6 %)	84 (46.4 %)	4 (3.6 %)	54 (49.5 %)
Odds ratio	3.364*		6.171*	

* mean *P* < 0.05

The MCB tests in the two age groups demonstrated differences in sensitivity, specificity, and PPV (*p* < 0.05). The MCB tests also demonstrated improved sensitivity (80 %) and NPV (87.5 %) for elderly patients, and higher specificity (84.9 %) and PPV (55.6 %) for younger patients. Cardiac biomarkers exhibited a higher positive likelihood ratio (LR+) in the diagnosis of acute cardiovascular syndrome in young patients (3.49 vs. 1.43) (Table 4).

**Table 4:** Diagnostic Capabilities of MCB tests by Age

	Patient of Age		*P* value
	Patients ≥65 years	Patients <65 years	
Sensitivity	80.0 % (64.1–90.0)	52.6 % (37.3–67.5)	<0.05^*^
Specificity	44.1 % (35.3–53.4)	84.9 % (76.9–90.5)	<0.05^*^
PPV	31.1 % (22.5–41.3)	55.6 % (39.6–70.5)	<0.05^*^
NPV	87.5 % (76.4–93.8)	83.3 % (75.2–89.2)	0.28
LR+	1.43 (1.13–1.81)	3.49 (2.02–6.00)	
LR−	0.45 (0.22–0.91)	0.56 (0.39–0.79)	

PS: PPV, NPV, LR+ and LR− mean positive and negative predictive values, positive and negative likelihood ratios, respectively

*mean *P* < 0.05

Differences were also observed in the sensitivity and PPV of MCB tests across genders. The biomarkers had higher sensitivity (80 %) and NPV (93.1 %) in female patients, while the specificity (66.1 %) and PPV (43.4 %) were higher in male patients. The LR+ diagnosis of acute coronary syndrome was higher in female patients than in male patients (2.03 vs. 1.81) (Table 5).

**Table 5:** Diagnostic Capabilities of MCB tests by Gender

	Patient of gender		*P* value
	Men (*N* = 181)	Women (*N* = 109)	
Sensitivity	61.1 % (47.8–73.0)	80.0 % (58.4–91.9)	<0.05^*^
Specificity	66.1 % (57.5–73.8)	60.7 % (50.3–70.2)	0.23
PPV	43.4 % (32.9–54.6)	31.4 % (20.3–45.0)	0.07
NPV	80 % (71.4–86.5)	93.1 % (83.6–97.3)	<0.05^*^
LR+	1.81 (1.31–2.49)	2.03 (1.45–2.85)	
LR−	0.59 (0.41–0.84)	0.33 (0.14–0.81)	

PS: PPV, NPV, LR+ and LR− mean positive and negative predictive values, positive and negative likelihood ratios, respectively

*mean *P* < 0.05

When the efficacy of MCB tests across both age and gender groups was investigated, MCB tests showed the highest sensitivity (87.5 %) and negative likelihood ratio (LR−) in elderly female patients, and the highest specificity (91.2 %), NPV (93.9 %), and LR+ (5.67) in young female patients (Table 6).

**Table 6:** Diagnostic Capabilities of MCB tests by Gender and Age

	≥65 years old (*N* = 146)		<65 years old (*N* = 144)	
	Male (*N* = 75)	Female (*N* = 71)	Male (*N* = 106)	Female (*N* = 38)
Sensitivity	73.7 % (51.2–88.2)	87.5 % (64–96.5)	52.9 % (36.7–68.5)	50.0 % (15.0–85.0)
Specificity	46.4 % (34.0–59.3)	41.8 % (29.7–55.0)	81.9 % (71.5–89.1)	91.2 % (77.0–97.0)
PPV	31.8 % (20.0–46.6)	30.4 % (19.1–44.8)	58.1 % (40.8–73.6)	40.0 % (11.8–76.9)
NPV	83.9 % (71.1–89.2)	92 % (75.0–97.8)	78.7 % (68.1–86.4)	93.9 % (80.4–98.3)
LR+	1.37 (0.96–1.98)	1.50 (1.12–2.01)	2.93 (1.63–5.26)	5.67 (1.32–24.37)
LR−	0.57 (0.25–1.26)	0.29 (0.08–1.13)	0.57 (0.39–0.83)	0.55 (0.20–1.47)

PS: PPV, NPV, LR+ and LR− mean positive and negative predictive values, positive and negative likelihood ratios, respectively

## Discussion

Acute coronary syndrome is diagnosed on the basis of typical angina, electrocardiogram (ECG) results, and cardiac enzymes analysis. Patients with positive for two out of the three clinical criteria are diagnosed with acute coronary syndrome. This study focused on patients who experienced chest pain and who were suspected of having acute coronary syndrome. If the patient demonstrated ST-segment elevation and LBBB they were diagnosed with acute coronary syndrome. In such cases, cardiac enzyme analysis was not necessary. Patients who experience chest pain without any obvious change in their electrocardiogram or patients with atypical symptoms such as chest tightness, fainting spells, sweating, and shortness of breath are confounding for clinicians. In such cases, it is necessary to examine cardiac enzymes to aid in arriving at the proper diagnosis. Thus, we investigated the diagnostic efficacy of MCB tests in patients suspected of having acute coronary syndrome.

Rapid and accurate recognition together with efficient or rapid service strategy (e.g. same-day transfer) had been shown to increase the access rate to subsequent cath-lab management and further reduced hospital stay length in ACS patients. Based on this, an efficient and cost-effective diagnostic strategy prior to optimal implementation of health care model had become a major challenge in current clinical practice. For example, Campo et al. proposed a Hub and Spoke model in intermediate-to-high risk non-ST-segment elevation ACS patients referral, while other center ever reported [9]. Previous studies have demonstrated the effects of age and gender on the diagnosis of acute coronary syndrome. The use of electrocardiograms in elderly patients can be problematic, such as in cases when the patient has a pacemaker or relatively low ST-segment elevation [10]. Elderly patients can exhibit symptoms similar to myocardial infarction, such as with gastritis or pneumonia [11–13]. Further, only a small proportion of elderly patients with acute coronary syndrome experience typical angina symptoms [14]. Finally, patients with congestive heart failure, diabetes, or coronary artery bypass graft surgery may exhibit atypical chest pains [14]. The current study also demonstrated that elderly patients are likely to exhibit these atypical factors. In addition, patients who smoke or have dyslipidemia exhibit relatively typical chest pain symptom, leading to many cases of acute coronary syndrome in younger patients [15].

Conventionally, female patients are less likely to seek for medical help than male patients. In addition, the symptoms of myocardial ischemia with chest pain are apparent atypically and easily confused with female chest wall or breast lesions with pain [15]. A relatively low proportion of electrocardiograms in women show ST-segment elevation; thus, the use of biomarkers to analyze the probability of acute coronary syndrome in women can help overcome these difficulties in a more efficient manner. This study also demonstrated that young and female patients have a relatively higher risk of acute coronary syndrome after positive biodetection.

Troponin I is an important biomarker for diagnosing acute myocardial infarction due to its sensitivity and specificity in the detection of cardiomyocyte injury, as well as its character of remaining detectable in the blood for a continuous period of 4 days, and up to 2 weeks. However, there is also a shortcoming for clinical Troponin I use, for example, it only begins to rise 6 h after disease onset. However, patients with acute coronary syndrome will often seek for medical attention shortly after symptoms emerge, resulting in a time delay between the clinical symptoms and diagnostic results. McCord’s et al. has reported that 49 % of patients with acute coronary syndrome arrive at the hospital within 4 h of disease onset, leading to a large number of patients remain undiagnosed in the emergency room for hours due to the time gap as mentioned above [16].

According to a joint European Society of Cardiology (ESC) and American College of Cardiology (ACC) committee recommendation for diagnostic indicators of myocardial infarction, the 99th percentile upper range of troponin I in normal population serves as the recommended critical value for a diagnosis of myocardial infarction and myocardial damage; however, this may cause complications for an accurate diagnosis [17]. Although troponin I demonstrates high specificity, several factors, including renal failure, sepsis, chest trauma, and anthracycline use, may generate false-positive results due to elevated troponin I levels, resulting in erroneous treatment and unnecessary hospital stays [18].

In the current study MCB tests had higher specificity in elderly women than in men (Table 6). In particular, troponin I showed high specificity in female patients, which may help to clarify the real disease in a great portion of women manifested as angina clinically. In this regard, if a patient presented with abnormal troponin *I* value, the possible misclassification as normal or false negative rate in women can be largely avoided and can be discharged from ED safely. Studies have shown that if only cardiac biomarkers are monitored within the first 6 h of onset of myocardial infarction, they are unable to provide sufficient diagnostic capability in terms of sensitivity and specificity [17]. Due to these aforementioned reasons, patients are unable to receive accurate diagnoses in a timely fashion, which not only results in overcrowded emergency rooms but also causes unnecessary hospital stays and significantly higher costs [18]. Of the 290 patients recruited in this study, 18.3 % of the women experienced cardiovascular events, which is considerably lower than the 29.3 % of men who experienced a cardiovascular event. Previous studies have demonstrated that the proportion of women with abnormal troponin I levels was relatively low because only approximately 6 % of troponin T and 3 % of troponin I exist in a free-floating state within the myocardial tissue of female gender. Since men typically have a greater number of cardiomyocytes than women, therefore the troponin I levels in men are easier to monitor or identified [19].

From the perspective of emergency room doctors, a quick and accurate diagnosis of acute coronary syndrome in elderly patients with chest pain is a major challenge. While patients’ medical records and physiology can be conveniently examined, both these methods have lower sensitivity and specificity. Although electrocardiograms are continuously monitored and in general may have a diagnostic specificity of 92 %, their sensitivity is only 39 % [20]. Several studies have shown that the use of MCB tests—such as troponin I, CK-MB, and myoglobin, which are related to cardiomyocyte necrosis, or BNP, which is related to cardiomyocyte function—can effectively improve the risk classification of patients with acute coronary syndrome. Due to the traditional shortcomings of individual biomarkers, the American College of Cardiology recommends that myoglobin be included in our MCB approach to improve diagnostic capability [21]. Since myoglobin is commonly present in skeletal muscle and myocardium, its levels will elevate 1–2 h after ACS symptoms. While myoglobin has a relatively low specificity, it has a very high sensitivity. Thus, if myoglobin is added as a biomarker for the identification of acute coronary syndrome, the diagnostic workflow may be executed more quickly and accurately. Of note, the proportion of female gender varied largely in different age groups (categorized as <, or > = 65 years old) in our current work, which may potentiate the bias in the results generated.

By using a MCB approach we were able to effectively identify patients with low-risk chest pain. In particular, the MCB approach demonstrated excellent diagnostic capability in female and elderly patients, both of whom are considered to be challenging in accurate diagnosis. In our current work, both populations presented with relatively good sensitivity and NPV (> = 80 %). In particular, the sensitivity and specificity for elderly female patients were near 90 %. In light of these findings, we can not only reduce the amount of time patients spend in the emergency room but also lessen the burden on attending clinicians.

One study showed that when 1285 emergency room chest pain patients were monitored for myoglobin, CK-MB, and troponin I over 90 min, they achieved a NPV of 100 %. Of the 508 patients who were diagnosed as negative, only one returned to the emergency room with myocardial infarction 30 days after the diagnosis [22]. This demonstrates that multiple biomarker tests can effectively exclude patients with chest pains and potential acute coronary syndrome. Furthermore, non-myocardial infarction patients can be excluded when the elevation of the CK-MB level over 2 h exceeds 1.5 ng/mL or the troponin I level exceeds 0.2 ng/mL [23].

The current study demonstrates that a MCB approach is more effective in the evaluation of elderly patients than young patients. During the process of assessing and selecting potential groups and excluding potential cases, the elderly patient group showed high NPV (87.5 %) and sensitivity (80 %), both of which were statistically significant. These results showed that by using the MCB approach for elderly patients with chest pain, one can quickly and accurately exclude acute coronary syndrome and other cardiovascular diseases in 2 h. Thus, the amount of time patients spend in the emergency room is reduced and unnecessary hospital stays are avoided. A recent study showed that a MCB approach can reduce the amount of time chest pain patients spend in the emergency room from 1 to 2 days to just 3–4 h [24]. Another study shows that by expediting the diagnostic process, the MCB approach can also lessen the costs of treatment for patients with chest pain [25]. Thus, this tool may aid in reducing emergency room crowding and minimize subsequent medical expenses [25].

Moreover, by using point-of-care monitoring MCB, we can speed up report completion time and therefore improve the efficacy of clinical practice. Studies have shown that as compared with traditional testing, the reporting time in point-of-care monitoring systems can be decreased by 75–85 % [26]. Such point-of-care monitoring systems are able to aid in expediting diagnosis.

## Conclusions

Our results revealed that due to the high sensitivity and NPV of MCB, the clinical implementation of such diagnostic tools can help physicians exclude acute coronary syndrome quickly and accurately in elderly patients present with atypical chest pain in the emergency department. Furthermore, we also demonstrated that MCB tests are effective in the diagnosis of acute coronary syndrome in elderly and female patients. The clinical use of MCB in high-risk, atypical chest pain or in elderly patients can therefore facilitate accurate and proper diagnosis in a more timely fashion, reduce the time spent in the emergency room, and expedite treatment delievery. Finally, MCB tests may enormously improve the clinical workflow of emergency treatment and effectively reduces the rates of diagnostic misclassification encountered in certain challenging patient populations from the emergency room.

## Abbreviations

ACS: Acute coronary syndrome
BNP: Brain natriuretic peptide
CK-MB: Creatine kinase-myocardial band isoenzyme fraction
ECG: Electrocardiogram
ED: Emergency department
LBBB: Left bundle branch block
MCB: Multiple cardiac biomarker
NSTEMI: Non-ST-segment elevation myocardial infarction
POCT: Point-of-care testing
STEMI: ST-segment elevation myocardial infarction
